# Copy number and gene expression differences between African American and Caucasian American prostate cancer

**DOI:** 10.1186/1479-5876-8-70

**Published:** 2010-07-22

**Authors:** Amy E Rose, Jaya M Satagopan, Carole Oddoux, Qin Zhou, Ruliang Xu, Adam B Olshen, Jessie Z Yu, Atreya Dash, Jerome Jean-Gilles, Victor Reuter, William L Gerald, Peng Lee, Iman Osman

**Affiliations:** 1 Department of Urology, New York University School of Medicine, New York, New York 10016, USA; 2 Department of Epidemiology and Biostatistics, Memorial Sloan-Kettering Cancer Center, New York, New York 10065, USA; 3 Department of Pediatrics, New York University School of Medicine, New York, New York 10016, USA; 4 Department of Surgery, Memorial Sloan-Kettering Cancer Center, New York, New York 10065, USA; 5 Department of Pathology, New York University School of Medicine, New York, New York 10016, USA; 6 Department of Pathology, Memorial Sloan-Kettering Cancer Center, New York, New York 10065, USA

## Abstract

**Background:**

The goal of our study was to investigate the molecular underpinnings associated with the relatively aggressive clinical behavior of prostate cancer (PCa) in African American (AA) compared to Caucasian American (CA) patients using a genome-wide approach.

**Methods:**

AA and CA patients treated with radical prostatectomy (RP) were frequency matched for age at RP, Gleason grade, and tumor stage. Array-CGH (BAC SpectralChip2600) was used to identify genomic regions with significantly different DNA copy number between the groups. Gene expression profiling of the same set of tumors was also evaluated using Affymetrix HG-U133 Plus 2.0 arrays. Concordance between copy number alteration and gene expression was examined. A second aCGH analysis was performed in a larger validation cohort using an oligo-based platform (Agilent 244K).

**Results:**

BAC-based array identified 27 chromosomal regions with significantly different copy number changes between the AA and CA tumors in the first cohort (Fisher's exact test, P < 0.05). Copy number alterations in these 27 regions were also significantly associated with gene expression changes. aCGH performed in a larger, independent cohort of AA and CA tumors validated 4 of the 27 (15%) most significantly altered regions from the initial analysis (3q26, 5p15-p14, 14q32, and 16p11). Functional annotation of overlapping genes within the 4 validated regions of AA/CA DNA copy number changes revealed significant enrichment of genes related to immune response.

**Conclusions:**

Our data reveal molecular alterations at the level of gene expression and DNA copy number that are specific to African American and Caucasian prostate cancer and may be related to underlying differences in immune response.

## Background

African Americans (AA) have a higher incidence of prostate cancer (PCa) and a higher mortality from the disease compared to age-matched Caucasians (CA)[1-4]. It remains controversial, however, whether these inequalities are solely attributable to socio-economic variables or if genetic and/or molecular differences also play a significant role [5-10]. We previously reported that between 1990 and 2000, the disparity between racial groups with regard to both pathologic stage and age at RP diminished significantly among patients treated at the Manhattan Veteran's Hospital, an equal access to care institution[11]. Disparity in Gleason score, however, a characteristic believed to be more reflective of tumor biology and less reflective of screening efforts, remained stable over the same period of time. Our data also suggest that socioeconomic factors play a limited role in PSA recurrence among AA men treated with RP[12]. Both of our investigations as well as those by other groups showing differences in gene expression and single nucleotide polymorphisms in genes related to the androgen receptor[13-16], growth factors[17-19], and apoptosis[20] support the possibility that disparities in outcome between AA and CA PCa patients may have an underlying molecular or genetic component.

Molecularly targeted, patient-specific therapy applied earlier in the disease course has the potential to improve survival for both AA and CA PCa patients. The development of such therapies, however, first requires an accurate characterization of the molecular pathways involved in tumorigenesis. If the observed racial disparities in PCa are the result of distinct alterations in tumor biology, it follows that the appropriate molecular target for each group may be different. An improved understanding of these alterations is a prerequisite for the development of effective, patient-specific, molecularly targeted therapy for both patient groups.

We examined both DNA copy number changes and gene expression profiles in a cohort of AA and CA PCa patients using BAC-based array comparative genomic hybridization (aCGH), oligo-based aCGH, and gene expression array. Our goal was to identify AA/CA-specific changes in DNA copy number and mRNA expression that might contribute to the relatively aggressive phenotype associated with AA prostate cancer. Using this genome-wide approach, we identified distinct regions of DNA copy number gain and loss in AA versus CA tumors, a subset of which were validated in a larger, independent cohort. The altered DNA copy changes were concordant with gene expression, and thus may be of particular biologic relevance. Our results suggest that molecular differences may contribute to PCa health disparities.

## Methods

### Patient population

The DNA copy number analyses consisted of PCa patients (n = 41) treated with radical prostatectomy (RP) at Memorial Sloan-Kettering Cancer Center (MSKCC, New York, NY). Twenty AA patients were frequency matched with 21 CA patients for age, PSA, stage and Gleason score to the extent possible. Gene expression profiling was also performed on 33 tumors from this same cohort (RNA isolated from 19 AA and 14 CA passed the QC for array hybridization). The study was approved by the Institutional Review Board of MSKCC.

### Sample evaluation

Prostatic tissues were obtained from RP specimens performed as part of routine clinical management at MSKCC. Tissues were snap-frozen in liquid nitrogen and stored at -80°C. Samples were examined using hematoxylin and eosin-stained cryostat sections. An experienced genitourinary pathologist (WLG) manually dissected non-neoplastic tissue. Samples included for analysis contained 60-80% PCa cell nuclei.

### BAC-based aCGH

The Spectral Chip 2600 (Spectral Genomics Houston, TX), a BAC-based array CGH platform, was used to identify chromosomal alterations in the first cohort of tumors (AA = 20, CA = 21). Genomic DNA was extracted from OCT-embedded specimens as previously described[21]. Karyotypically normal female DNA was used as the reference DNA (Promega, Madison, WI). Restriction and labeling of DNA was performed by Spectral Genomics according to manufacturer protocol. Briefly, 2 μg of DNA was digested with *Eco*RI or *Dpn*II (10 U/μg) at 37°C for 16 hours. DNA was purified and each sample separately labeled with cyanine-5 (Cy5) and cyanine-3 (Cy3) dCTPs. Labeled test and reference DNAs were mixed, co-precipitated with isopropanol, washed, and resuspended in hybridization solution. DNA mixtures were denatured at 72°C for 10 minutes, prehybridized at 37°C for 30 minutes, and co-hybridized to the arrays with cover slips for 16 or more hours at 37°C. All clones were represented on the respective array in duplicate.

### Oligo-based aCGH

As part of a separate ongoing study of 28 AA and 180 CA patients at MSKCC, aCGH was performed using the Agilent 244K oligonucleotide array containing 244,000 probes with an average spatial resolution of ~ 9 kb (Agilent Santa Clara, CA). As in the BAC-array, 2 μg of gDNA was labeled and hybridized to the array using the standard oligonucleotide aCGH protocol as per the manufacturer.

### Gene expression profiling

RNA was isolated from 19 AA and 14 CA tumors (from the initial cohort of 20AA and 21CA utilized for BAC-based aCGH) and hybridized to the Affymetrix HG-U133-Plus 2 arrays as per the manufacturer protocol. Array data were normalized using the robust multichip average (RMA).

### Statistical Analysis

The methodologies used for these analyses are briefly summarized below. The effective sample sizes for Steps 1 and 2 were 20 AA and 21 CA patients, while 19 AA and 14 CA patients were used for Step 3. Hierarchically clustering of the 19 AA and 14 CA patients was performed using the average linkage method.

#### Step 1: Identifying genome-wide copy number changes in each patient

Circular binary segmentation (CBS)[22] was used to segment the genome of each patient into regions having homogeneous copy number. These were classified into segments exhibiting copy number gain, normal copy number and copy number loss. For each tumor sample, the average and standard deviation of the segment intensities were obtained. Any segment having intensity exceeding (or smaller than) the average plus (or minus) 2*standard deviation was declared to have copy number gain (or loss). All other segments were declared to have normal copy numbers. The BAC array does not contain a dense set of probes, thus we divided the genome into 552 regions of 5 MB and examined the copy number change of each patient identified using CBS to record whether that region had a copy number gain, loss or normal copy number.

#### Step 2: Identifying noteworthy genomic regions exhibiting significant copy number differences in AA versus CA patients

In each 5 MB region, we considered a 3 × 2 table, with the rows representing number of patients with copy number gain, copy number loss or normal copy number in that region and the column representing AA and CA patients. We compared the copy number changes in the 20 AA versus 21 CA patients using a Fisher's exact test based on the 3 × 2 table in each region. We identified regions having p-values less than 0.05. Due to the exploratory nature of our analyses, we did not adjust the p-values for multiple comparisons and prioritized regions having p-value < 0.05 for further investigations.

#### Step 3: Investigating whether copy number gains or losses are associated with gene expression changes

In each noteworthy region, gene expression was compared between AA patients having copy number gain versus those with normal copy number, and those having copy number loss versus those with normal copy number. This analysis was conducted separately for the CA patients. A two-sample t-test was used for these analyses. Because the oligo-based arrays consist of a comprehensive set of genes covering a substantial part of the genome, we adjusted these analyses for multiple comparisons, and declared genes having adjusted p-values < 0.05 as statistically significant.

### Pathway and Gene Ontology (GO) Analyses

Functional annotation and pathway analysis of overlapping gene lists from significantly altered genomic segments were preformed using the DAVID Functional Annotation Tool and Database[23]. A modified, more conservative Fisher's Exact p-value, or EASE score, is used to determine if there is a significant level of enrichment in the gene set. An EASE score of P < 0.05 was considered significant using a minimum gene count threshold of ≥2 and an EASE threshold maximum probability ≤ 0.1.

## Results

Clinicopathologic variables for the initial cohort of 20AA and 21CA patients are presented in Table 1. The patients were frequency matched for age, PSA, Gleason score, and stage to the extent possible. In the initial cohort of patient specimens utilized for BAC-based aCGH, the profiles were similar between AA and CA with regard to age (mean 59 years both groups), PSA (mean 8.5AA; 8.3 CA), pathologic stage, and Gleason score (mean score = 7 in both groups).

**Table 1:** Baseline clinicopathologic variables of African American and Caucasian American patients and tumors utilized for BAC-based DNA copy number analysis and gene expression profiling

	African American(n = 20)	Caucasian American(n = 21)*
**Age (years)**		
50-54	7 (35%)	5 (24%)
55-59	6 (30%)	10 (48%)
60-64	2 (10%)	3 (14%)
≥65	5 (25%)	3 (14%)
		
**PSA**		
Mean, SD	8.5, 3.5	8.3, 4.0
Range	4-17	3-17
		
**Stage**		
II	12 (60%)	16 (76%)
≥III	8 (40%)	5 (24%)
		
**Gleason score**		
<7	3 (15%)	6 (29%)
=7	16 (80%)	11 (52%)
>7	1 (5%)	4 (19%)
		

*41 tumors were utilized for BAC array (AA = 20, CA = 21); 33 of the 41 (AA = 19, CA = 14) were also utilized for gene expression.

### BAC-based aCGH identified 27 significantly different regions of chromosomal alteration between AA and CA tumors

**Figure 1:**
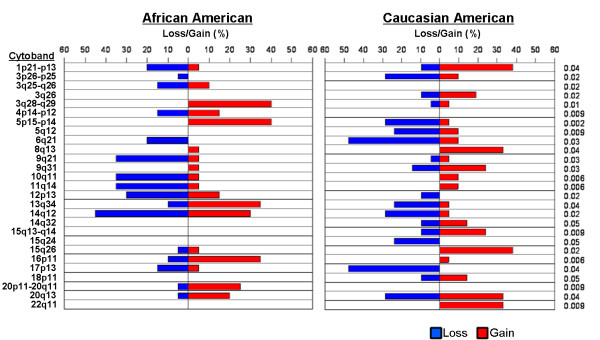
**BAC-based aCGH of 20 AA and 21 CA prostate tumors revealed 27 significantly altered genomic regions between the two groups**.

In the initial cohort of 20AA and 21 CA, BAC-based array CGH revealed 27 noteworthy regions that displayed differences in copy number variations between AA and CA tumors (Figure 1). Of these, 10 regions (3q25-q26, 3q28-q29, 4p14-p12, 9q21, 10q11, 11q14, 12p13, 14q12, 16p11, 20p11-20q11) were more commonly altered in AA patients compared to CA. 15 regions were more commonly altered in CA patients (1p21-p13, 3p26-p25, 3q26, 5q12, 6q21, 8q13, 9q31, 14q32, 15q26, 15q13-q14, 15q24, 17p13, 18p11, 20q13, 22q11), and 2 regions (5p15-p14 and 13q34) were significantly altered in both groups but in different directions. We did not observe any significant changes between the 2 groups on chromosome 2,7,19, or 21.

### oligo-based aCGH identified 23 significantly different regions of chromosomal alteration between AA and CA tumors

**Figure 2:**
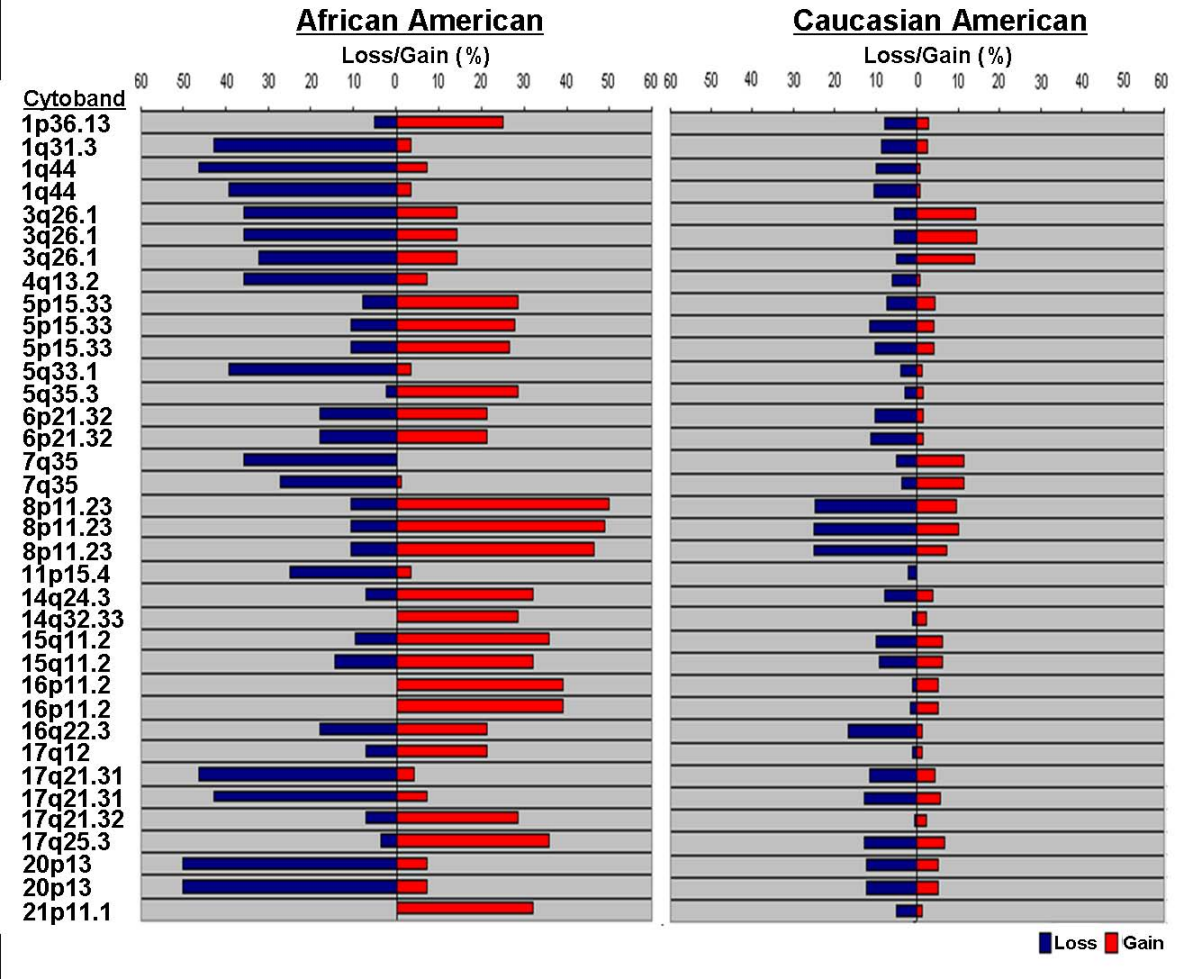
**Oligo-based aCGH of 28AA and 180CA prostate tumors revealed 23 unique chromosomal regions (represented by 36 probes) with significantly different (P ≤ 0.0001) DNA copy number**.

In the larger ongoing study utilizing oligo-based aCGH, a total of 579 genomic regions exhibited copy number gains or losses in at least 10% of the AA and CA samples. We then compared the copy number changes in AA versus CA patients in these 579 regions and ranked the regions by increasing order of the p-values. The 23 most significantly altered regions (represented by 36 probes) with P-value ≤ 0.0001 are shown in Figure 2. Of these regions, 9 were more commonly lost in AA patients compared to CA patients (1q31.3, 1q44, 3q26.1, 4q13.2, 5q33.1, 7q35, 11p15.4, 17q21.31, and 20p13), while 12 showed significant gains in AA compared to CA patients (1p36.13, 5p15.33, 5q35.3, 8p11.23, 14q24.3, 14q32.33, 15q11.2, 16p11.2, 17q12, 17q21.32, 17q25.3, and 21p11.1). Two regions, 6p21.32 and 16q22.3 had both significant gains and losses in AA patients compared to CA. As in the BAC-based analysis, we did not find significant genomic alterations in chromosomes 2 or 19.

Comparison of the 27 noteworthy identified using the BAC array with the 23 most significantly altered regions from the oligo-array revealed 4 chromosomal regions of overlap: 3q26 (narrowed to 3q26.1 in oligo array), 5p15-p14 (5p15.33 oligo), 14q32 (14q32.33 oligo), and 16p11 (16p11.2 oligo). Region 3q26 (3q26.1) showed significant losses in AA tumors compared to CA tumors using both platforms, while regions 5p15 (5p15.33) and 16p11 (16p11.2) showed significant gains in AA tumors compared to CA tumors in both analyses. Region 14q32 (14q32.33) showed significant gains in the CA tumors using the BAC-based platform, and significant gains in the AA tumors using the oligo-based platform.

### Gene expression profiling revealed distinct clustering of patients by racial group

**Figure 3:**
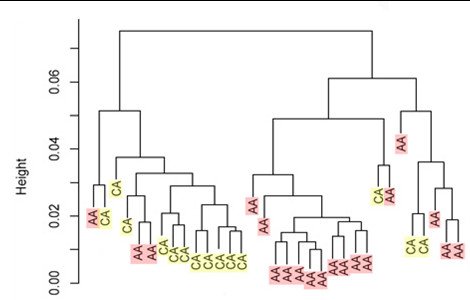
**Hierarchical clustering of 19 AA and 14 CA prostate tumors revealed distinct clusters, with only 3 tumors from each group that are misclassified**.

**Figure 4:**
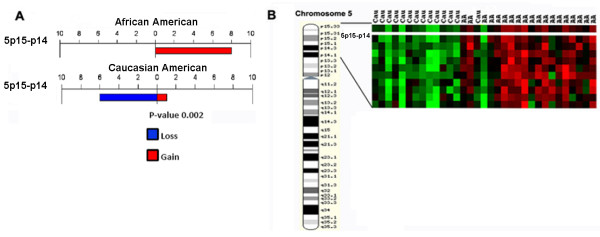
**Correlation between copy number gains in AA tumors (**A**) and overexpression of a subset of genes in AA tumors at 5p15-p14 (**B**)**.

Hierarchical clustering of 19 AA and 14 CA patients (from the original cohort of 20AA/21 CA) revealed two distinct clusters separating AA from CA tumors, with only 3 patients in each cluster who did not classify correctly into their respective group (Figure 3). To correlate gene expression with aCGH, we examined the expression patterns of the subset of genes located within the 27 noteworthy locations identified in the BAC-based aCGH analysis. One example of DNA/RNA correlation is represented in Figure 4A and 4B. As assessed using aCGH, cytolocation 5p15-p14 showed copy number gains in 8 African Americans and copy number loss in 6 Caucasian patients (Figure 4A). Expression analysis of the subset of genes located at 5p15-p14 revealed a distinct clustering of genes overexpressed in AA and underexpressed in CA tumors (Figure 4B) with only one tumor that appears to be misclassified. Thus, the gene expression profile showed concordance with the copy number data in that the genes from this region are predominantly overexpressed in AA but underexpressed in CA tumors. A similar pattern of correlation was observed in all of the 27 altered regions.

### Gene ontology and functional annotation of gene sets in the 4 regions of chromosomal overlap revealed over-representation of pathways related to immunity

**Figure 5:**
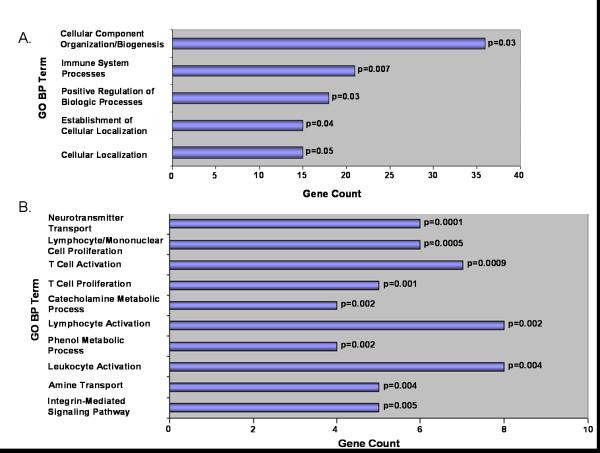
**Functional annotation analysis of genes contained within the 4 chromosomal regions that were significantly altered in both the BAC-based aCGH of AA and CA tumors (N = 41) and the oligo-based aCGH of the independent cohort (N = 208)**. Genes contained within regions 3q26.1, 5p15.33, 14q32.33, and 16p11.2 revealed significant enrichment of immune-related genes when ranked by both gene count (5A) and by p-value (5B).

Overlapping genes in the 4 chromosomal regions (3q26.1, 5p15.33, 14q32.33, 16p11.2) that were found to be among the most significantly altered between AA and CA in the initial cohort of 41 tumors and in the validation cohort of 208 tumors showed significant enrichment of immunology-related Gene Ontology (GO) Biologic Process (BP) terms. When ranked by gene count, GO BP Term *Immune System Processes *was the second most enriched term with a total count of 21 genes from our set, representing 9% of the total number of genes annotated for the term (p = 0.007, Figure 5A). When ranked by p-value, the most significantly enriched terms were neurotransmitter transport (p = 0.0001), followed by lymphocyte/mononuclear cell proliferation (p = 0.0005), T cell activation (p = 0.0009), and T cell proliferation (p = 0.001)(Figure 5B). Other significantly enriched immunology-related GO BP Terms included lymphocyte activation (p = 0.002), leukocyte activation (p = 0.004), and integrin-mediated signaling pathways (p = 0.005).

## Discussion

The existence of racial disparities in prostate cancer is generally acknowledged, but the predominant factor influencing these disparities remains contested. Some believe that socioeconomic variables are primarily responsible for the worse outcome in AA PCa patients[24], while others recognize the possibility of biologic heterogeneity in AA versus CA tumorigenesis [8,11,12]. In the current study, we utilized an integrated genome wide approach to demonstrate that AA and CA prostate tumors exhibit molecular differences with regard to DNA copy number and gene expression. Thus, it is possible that AA and CA tumors harbor distinct areas of genomic instability or sensitivity to selective pressures that results in characteristic DNA copy number alterations. This instability may represent an inherited source of differential risk or a differential response to environmental factors between the two groups that might influence the outcome of disease.

The results of our integrated genomic analyses are consistent with those from two previous studies that identified molecular differences between AA and CA prostate tumors using gene expression profiling[25,26]. Our study makes the additional finding that DNA copy number alterations are a likely mechanism for these observed differences in gene expression. The gene expression study by Wallace and colleagues of 69 tumors from AA and CA patients revealed a relatively short list of 162 transcripts differentially expressed between the two cohorts[26]. Further analysis resulted in the creation a two-gene classifier (*CRYBB2 *and *PSPHL*) that was able to accurately separate AA from CA, although the role of these two genes as drivers of tumorigenesis in AA or CA is unclear at the present time. Another study of gene expression differences between AA and CA tumors identified cell death regulatory protein *TCEAL 7 *as differentially overexpressed in CA versus AA tumors[25]. This finding led authors to speculate that *TCEAL 7 *may play an oncosuppressive role that contributes to the relatively aggressive nature of PCa in AA.

Functional annotation and pathway analysis of genes mapping to the 4 genomic regions of overlap in our two independent cohorts revealed significant enrichment for ontologic annotations related to immune function. Included among the genes annotated as *Immune System Processes *were: *IL-27*, *ITGAL*, *ITGAM*, *ITGAD*, *IGHM*, *SPN*, *LAT*, and *AKT-1*. It is notable that two other published, independent gene expression profiling studies also noted enrichment of immune-related genes in their comparison of AA and CA tumors [25,26]. Specifically, immunoglobulin heavy constant mu (*IGHM*), which maps to 14q32.33, was one of the top 20 genes with higher expression in AA compared to CA tumors in the study by Wallace[26]. The list of differentially expressed genes reported in the Reams study showed significant enrichment of pathways related to interleukins[25]. Taken together, data suggest that differences in host immunity may influence the natural history of PCa in AA and CA patients, and our results show that these differences are likely present in the cancer genome.

These findings are particularly relevant in light of the recent emergence of immunotherapy as a potential treatment for PCa. A dendritic cell vaccine has gained approval by the Food and Drug Administration (FDA) for use in hormone refractory metastatic prostate cancer patients, and the first phase I trial of a hybrid peptide vaccine as adjuvant therapy for metastatic and non metastatic patients with was recently completed[27,28]. Based on our genomic analysis of AA and CA tumors, it is possible that AA and CA patients might respond differently to immuno-based therapies. As the use of immunotherapy expands to include a larger population of both primary and metastatic PCa patients, it will be important to consider how differences in host immunity might influence the response to therapy or the molecular readouts of treatment activity such as T cell proliferation.

The large range of chromosomal alterations observed in solid tumors have in the past made it difficult to identify a signature of alterations that are common in prostate cancer in the way that characteristic changes have been identified in lymphoid malignancies. Without such a signature, there is no basis for devising molecular targets for treatment, diagnosis, or prognostication that can be consistently used for specific groups of patients. It is noteworthy that in our study, 4 genomic regions were reproduced in an independent group of tumors using a different platform. Two of these regions (5p15.33 and 16p11.2) have been previously reported as common areas of genomic gain in prostate cancer. In one series of 18 prostate cancer cell lines and xenografts, 39% of samples had copy number gain at 5p15.33 and 39% had gains at 16p12.2-p11.2[29]. As in our study, the authors were able to demonstrate concordance between copy number gain and gene overexpression, most notably in genes mapping 16p12.2-p11.2 (*RBBP6, RGS11, and RABEP2). RABEP2 *maps to 16p11.2 and is a GTPase binding effector protein that has not been previously associated with PCa. The finding of copy number gains at 16p11.2 and overexpression of *RABEP2 *in this previous study of PCa cell lines and in our current study of human PCa tissues is reassuring of the validity of the data.

Both array CGH and gene expression arrays are methodologies with relatively high false positive rates. Correlation of DNA copy number and gene expression data enables one to filter out many false positive results and provides a basis for correlating gene expression changes with a specific altered genomic mechanism. In this regard, we report a high concordance between DNA copy number and gene expression in all of the 27 most significantly altered genomic regions between AA and CA prostate tumors. Lower concordance rates observed in other studies[30] may reflect differences in the regulation of expression of the genes observed in those studies or may be reflective of the greater difficulty inherent in working with RNA leading to artifacts. In our study, we prioritized sets of genes for pathway analysis based on the chromosomal regions that differentially affected AA and CA tumors in two independent patient cohorts. Of note, 14q32 was gained in CA patients in the initial cohort but gained in AA patients in the validation cohort. This discrepancy might be due to differences in the resolution and genomic region coverage of the BAC-based and oligo-based array platforms. It is possible that the BAC array missed the more focal copy number gain detected in the AA tumors by oligo-array. The published data showing that *IGHM*, which maps to 14q32.33, is significantly overexpressed in AA tumors[26] lend support to our oligo-based array finding that 14q32.33 shows significant copy number gains in AA tumors.

In conclusion, our study reveals molecular differences that characterize AA and CA PCa tumorigenesis. Pathway analysis revealed significant over-representation of inflammation and immunobiology-related genes. Further studies are warranted to adequately assess the clinical implications of these observed differences.

### Disclosures

The authors confirm that there are no conflicts of interest.

## Authors' contributions

AR participated in data analysis and wrote the manuscript. JS supervised the statistical analysis. CO participated in study design, data analysis, and drafting of the manuscript. QZ participated in the statistical analysis. RX performed experimental assays. AO participated in the statistical design of the study. JY participated in data analysis and drafting of the manuscript. AD was involved in the conceptual design of the study and drafting of the manuscript. JG participated in data analysis. VR participated in study design and interpretation of data. WG was involved in the study design and supervised all experiments. PL and IO served as the principal investigators. All authors read and approved the final manuscript.

## Abbreviations List

PCa: prostate cancer; AA: African American; CA: Caucasian American; RP: radical prostatectomy; CGH: comparative genomic hybridization; aCGH: array comparative genomic hybridization; BAC: bacterial artificial chromosome; MSKCC: Memorial Sloan-Kettering Cancer Center; CBS: circular binary segmentation; GO: gene ontology; BP: biologic process
